# A Turntable Setup for Testing Visual and Tactile Grasping Movements in Non-human Primates

**DOI:** 10.3389/fnbeh.2021.648483

**Published:** 2021-05-25

**Authors:** Daniela Buchwald, Stefan Schaffelhofer, Matthias Dörge, Benjamin Dann, Hansjörg Scherberger

**Affiliations:** ^1^Neuroscience Laboratory, Deutsches Primatenzentrum GmbH, Göttingen, Germany; ^2^Faculty of Biology and Psychology, University of Goettingen, Göttingen, Germany

**Keywords:** electrophysiology, multi-sensory, non-human primate, grasping, object interaction

## Abstract

Grasping movements are some of the most common movements primates do every day. They are important for social interactions as well as picking up objects or food. Usually, these grasping movements are guided by vision but proprioceptive and haptic inputs contribute greatly. Since grasping behaviors are common and easy to motivate, they represent an ideal task for understanding the role of different brain areas during planning and execution of complex voluntary movements in primates. For experimental purposes, a stable and repeatable presentation of the same object as well as the variation of objects is important in order to understand the neural control of movement generation. This is even more the case when investigating the role of different senses for movement planning, where objects need to be presented in specific sensory modalities. We developed a turntable setup for non-human primates (macaque monkeys) to investigate visually and tactually guided grasping movements with an option to easily exchange objects. The setup consists of a turntable that can fit six different objects and can be exchanged easily during the experiment to increase the number of presented objects. The object turntable is connected to a stepper motor through a belt system to automate rotation and hence object presentation. By increasing the distance between the turntable and the stepper motor, metallic components of the stepper motor are kept at a distance to the actual recording setup, which allows using a magnetic-based data glove to track hand kinematics. During task execution, the animal sits in the dark and is instructed to grasp the object in front of it. Options to turn on a light above the object allow for visual presentation of the objects, while the object can also remain in the dark for exclusive tactile exploration. A red LED is projected onto the object by a one-way mirror that serves as a grasp cue instruction for the animal to start grasping the object. By comparing kinematic data from the magnetic-based data glove with simultaneously recorded neural signals, this setup enables the systematic investigation of neural population activity involved in the neural control of hand grasping movements.

## 1. Introduction

Primate hands are versatile tools that are used in a variety of behaviors, starting from grasping objects to social interactions (Terry, 1970; Dunbar, 1991). More so, hand movements are easy to track and observe, making them a perfect candidate for trying to understand how our brain generates these fine and complex movements and reacts to the tactile and proprioceptive feedback the hand provides at the same time (Munk, 1890; Penfield and Boldrey, 1937; Jeannerod et al., 1995). For this reason, reach-to-grasp tasks provide valuable insights in movement generation and feedback processing.

To study the neuronal control of grasp movements, it is important that the animal executes a variety of grasp types and hand shapes, since behavior and neural activity can be properly correlated only then. In previous studies, this was often achieved by training the monkey to grasp a handle with different grasp types, usually a precision grip, where the object is hold between the index finger and thumb, and a power or side grip, where the object is clasped with the whole hand (Napier, 1962; Baumann et al., 2009). However, the low number of different grasps severely limits how well we can understand how the brain truly moves our hands. This led to different attempts of presenting a higher number of objects to the monkeys in order to get them to display a higher variety of different grasps. In different studies the objects are either presented on a multijoint manipulator (Sakata et al., 1995; Suresh et al., 2020) or even free hanging on strings (Vargas-Irwin et al., 2010). While this indeed allows to present a higher number of objects to the monkey it comes at the cost of a higher interaction with the animal, as the object is changed regularly. This means the experimenter either remains inside the setup or needs to step into it whenever a change is needed. Since most experiments aim to exchange the object after a few trials (ideally after every trial) this means a high downtime where the animal can not work and might be distracted.

We therefore wanted to automate this process, similarly as previously described by Murata et al. (2000), Raos et al. (2006), and Fattori et al. (2010). We used a round turntable with six compartments for different objects that could be rotated by computer control. To make usage of possible auditory cues harder, the rotation direction (left or right) was chosen at random. This allowed to present up to six objects automatically in random order, without having to switch manually between objects after each trial. Using this design, Schaffelhofer et al. (2015) were able to present to the monkey a total of 48 objects that were mounted on 8 turntables. The six objects on each turntable were then presented in random order until enough trials per object were collected, after which the turntable was manually exchanged. This way, the animal could work consistently and undisturbed by the experimenter, except for short breaks in the recording session when a turntable was switched.

In this paper, we present the current version of our turntable design. This setup features six objects on a turntable, allows for attention control of the animal, and instructs the animal to use visual or tactile object exploration to determine the appropriate grip type for each presented object. Improvements include a more accessible turntable plate to speed up the exchange process, a more precise detection whether an object is fully lifted, and projection of the cue LED for the animal directly onto the object to avoid an attention split. The latter is achieved by shining a very small red LED on a half-transparent mirror, giving the animal the impression as if the cue LED would sit directly onto the object without illuminating the object. We also present a new task paradigm where objects are not only presented visually, but also tactually, allowing to compare not only how the animal interacts with multiple objects, but also how it does so using different sensory information.

## 2. Materials and Methods

### 2.1. Animals

Here we present behavioral and neural data from one monkey that was trained on this setup. The monkey was a male, purpose-bred rhesus macaque (*Macaca mulatta*), that was born 2011 at the German Primate Center (Deutsches Primatenzentrum GmbH, Göttingen, Germany) and housed together with another male monkey with a 12 h dark-light cycle. Fluid intake through water bottles, the reward system (containing juice) or fruits and vegetables was monitored on training days, since fluids were used as main reward for successful trials. On days were the animal was not trained or recorded, he had free access to water. Access to food was never restricted. All experiments and housing were performed in accordance with European and German law and in agreement with the “Guidelines for the Care and Use of Mammals in Neuroscience and Behavioral Research” (National Research Council, 2003), as well as the NC3Rs “Guidelines for non-human primate accommodation, care and use” (National Centre for the Replacement, Refinement and Reduction of Animals in Research). Authorization for conducting this experiment was delivered by the Animal Welfare Division of the Office for Consumer Protection and Food Safety of the State of Lower Saxony, Germany (permit no. 14/1442 and 19/3132).

### 2.2. Implantation and Neuronal Signal Acquisition

**Figure 1:**
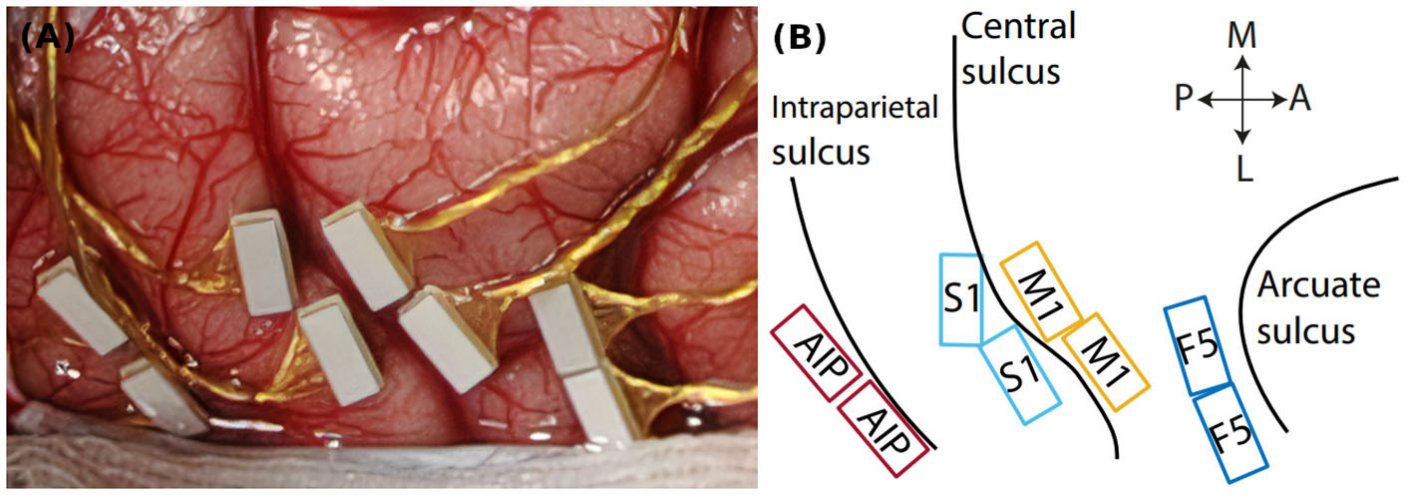
Cortical implantation sites. **(A)** Intrasurgical picture, showing the floating microelectrode arrays (white rectangles) implanted in parietal and frontal cortex. **(B)** Implantation schematic with implantation sites in parietal area AIP, somatosensory cortex (S1), primary motor cortex (M1), and premotor cortex (area F5). Black lines indicate cortical sulci. Double arrows indicate the medio-lateral (M-L) and posterior-anterior (P-A) direction.

To investigate the neuronal activity during the task, one animal was implanted with floating microelectrode arrays after training was completed (FMA, Microprobes for Life Sciences, Gaithersburg, MD, USA, see Musallam et al., 2007). Two arrays each were implanted into four different brain areas (see Figure 1): Anterior intraparietal cortex (AIP), primary somatosensory cortex (S1, area 3b), Primary motor cortex (M1) and premotor cortex (area F5). In this paper we present data from two example units from M1 and F5. For data acquisition two neural signal processors (Cerebus systems, Blackrock Microsystems Inc., Salt Lake City, UT, USA) were synchronized and connected to the implants. Data was recorded with 30 kHz and 16 bit and stored together with behavioral data on a hard drive for offline analysis (see Analysis methods, below).

### 2.3. Experimental Setup

In order to study how primates interact with different objects, a turntable setup was build that can automatically present up to six objects to the animals without human manual interaction. An earlier version of this setup has been employed in previous studies (Schaffelhofer, 2014; Schaffelhofer et al., 2015; Schaffelhofer and Scherberger, 2016).

**Figure 2:**
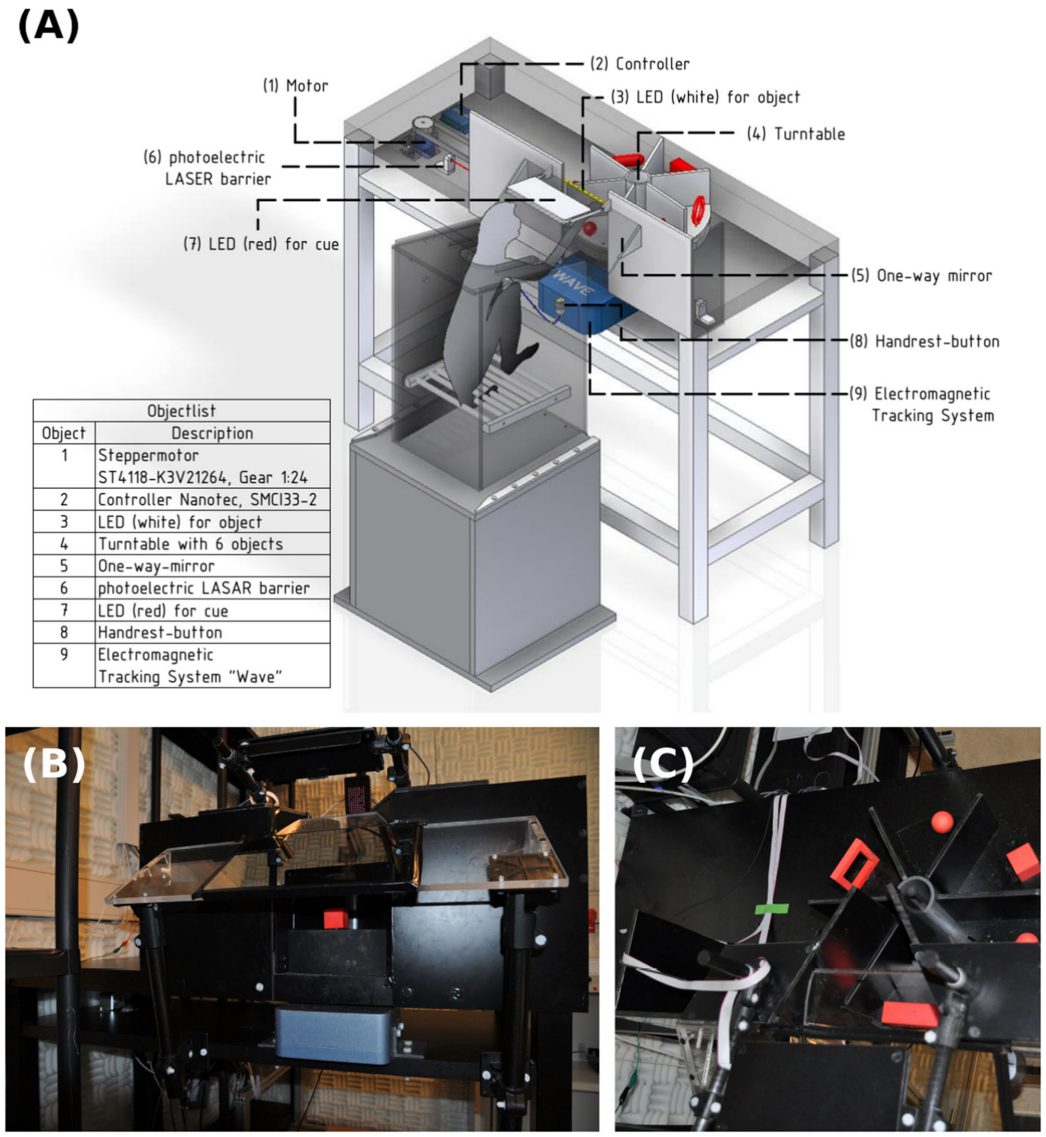
Overview over the experimental setup. **(A)** Monkey sitting in a primate chair on top of a plastic box. On the table an object plate (4) with six red objects that can be rotated by a motor (1) and controller (2) positioned underneath the table. The animal can only see and interact with one object at a time. A strip of white LED lights (3) is placed above the object for illumination. A photo-electric Laser barrier (6) is placed below the turntable and above the object counterweights, to detect the lifting of an object. A one-way mirror (5) is placed between the monkey and the table, which projects, from the animal's perspective, a red cue LED (7) onto the front object. A handrest button in front of the primate chair sets a consistent start position of the monkey's hand and an electromagnetic field generator of the hand tracking system is placed below the object plate to track the monkey's hand movements with a data glove (not shown). **(B)** Frontal view of the table. **(C)** Top view with turntable in the setup.

**Figure 3:**
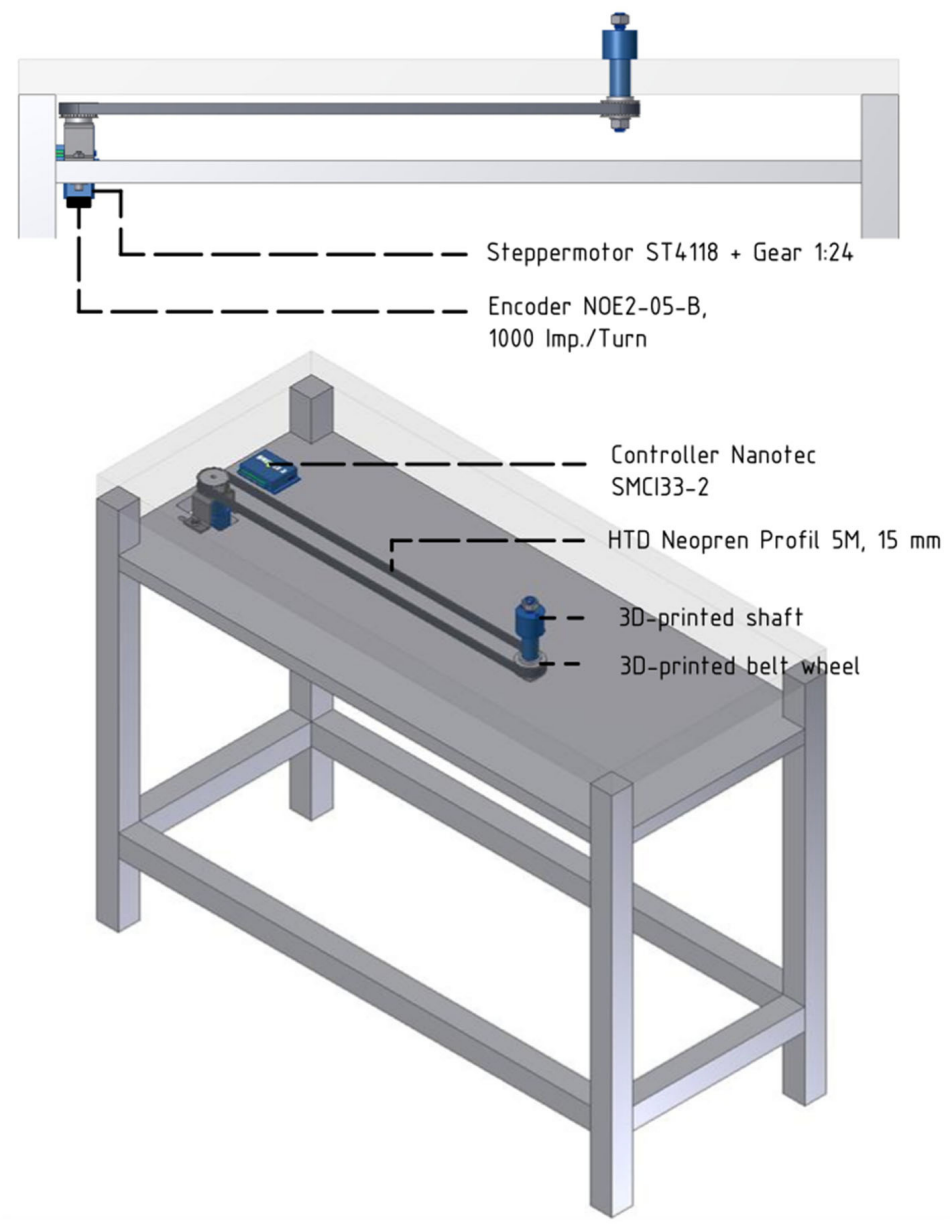
Overview over the motor control and belt system. Below the tabletop an extra level of the table houses the belt and motor system. The turntable (not shown) will be placed on top of the 3d-printed shaft and cogwheel, which fits the toothed belt connecting shaft and motor.

Core parts of the setup include a turntable, which is a round object plate featuring up to six 3D printed objects, a stepper motor (NEMA 17, Nanotec Electronic GmbH & Co. KG, Feldkirchen, Germany) that can rotate the turntable so that the selected object is presented to the front, as well as a stepper motor controller (SMCI33-2, Nanotec Electronic GmbH & Co. KG, Feldkirchen, Germany). These parts are mounted on a customized table so that the front object is reachable by the animal sitting in its primate chair (see Figure 2). The table fits the turntable plate on-top of a rotating axis (connected through two custom made carbon bolts that fit in two holes inside the turntable and axis) that fits a belt system connecting the turntable with the stepper motor (see Figure 3). In order to use the setup with a magnetic-based data glove (Schaffelhofer and Scherberger, 2012), the motor was positioned away from the turntable and connected to the turntable shaft (3D printed, Material: Nylon 12 [Versatile Plastic], Electro Optical Systems GmbH, Munich, Germany) with a toothed belt. Also, the setup was kept free of metal as much as possible with usage of plastic screws and fiberglass rods.

A capacitive-sensing touch button was fixed to the front of the primate chair, serving as a handrest button. The monkey was trained to place its hand on the button to initiate a trial. This ensured a defined start position of the hand for each trial and was also used as a safety measure during turntable rotation: the motor was programmed to move only when the button was pushed, ensuring an immediately stop of the rotating turntable should the monkey ever try to interfere with it. Relative position of the animal to the turntable was set by adjusting the height and position of a pedestal box supporting the primate chair.

**Figure 4:**
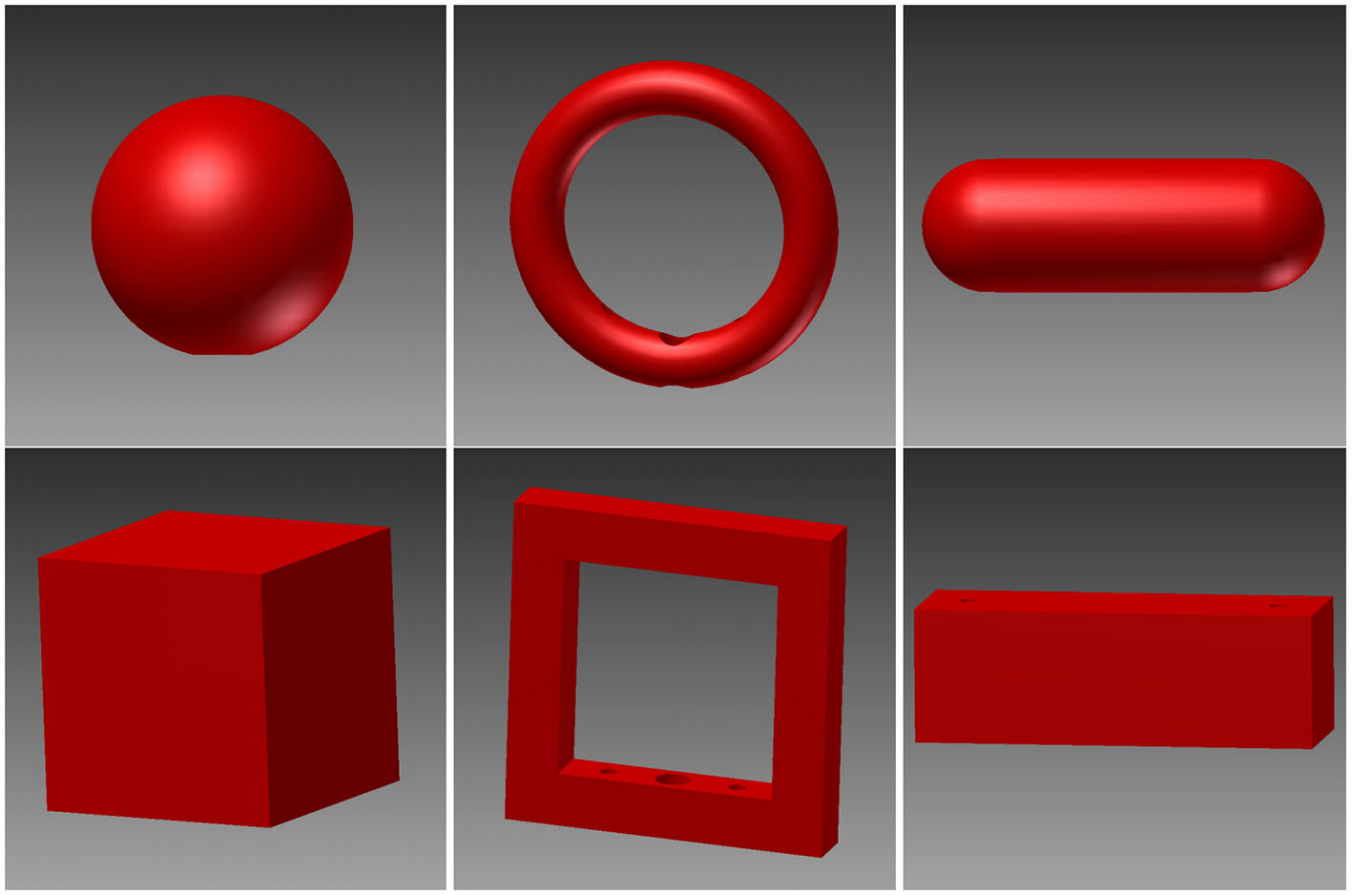
3D renders of the six objects. These objects were designed in pairs, where the two objects in the same column require a similar hand shape and grasp while looking and feeling differently. Top row: sphere, ring, and rounded bar. Bottom row: cube, edged version of the ring, and box.

For this study, six different objects were designed (see Figure 4): A sphere, a cube, a ring, a ring with edges, a bar and a bar with edges. These objects were designed to look and feel differently while each pair (each column in Figure 4) is grasped with a similar hand shape and grasp. The idea behind this design was to be able to disentangle the influence of sensory information and hand shape. Each object was designed in Autodesk Inventor (Autodesk, Inc.) and 3D printed out of plastic (PA 2200, Electro Optical Systems GmbH, Munich, Germany) by Shapeways (Shapeways Inc., New York, United States). We chose a red design for contrast to the black background of the turntable.

**Figure 5:**
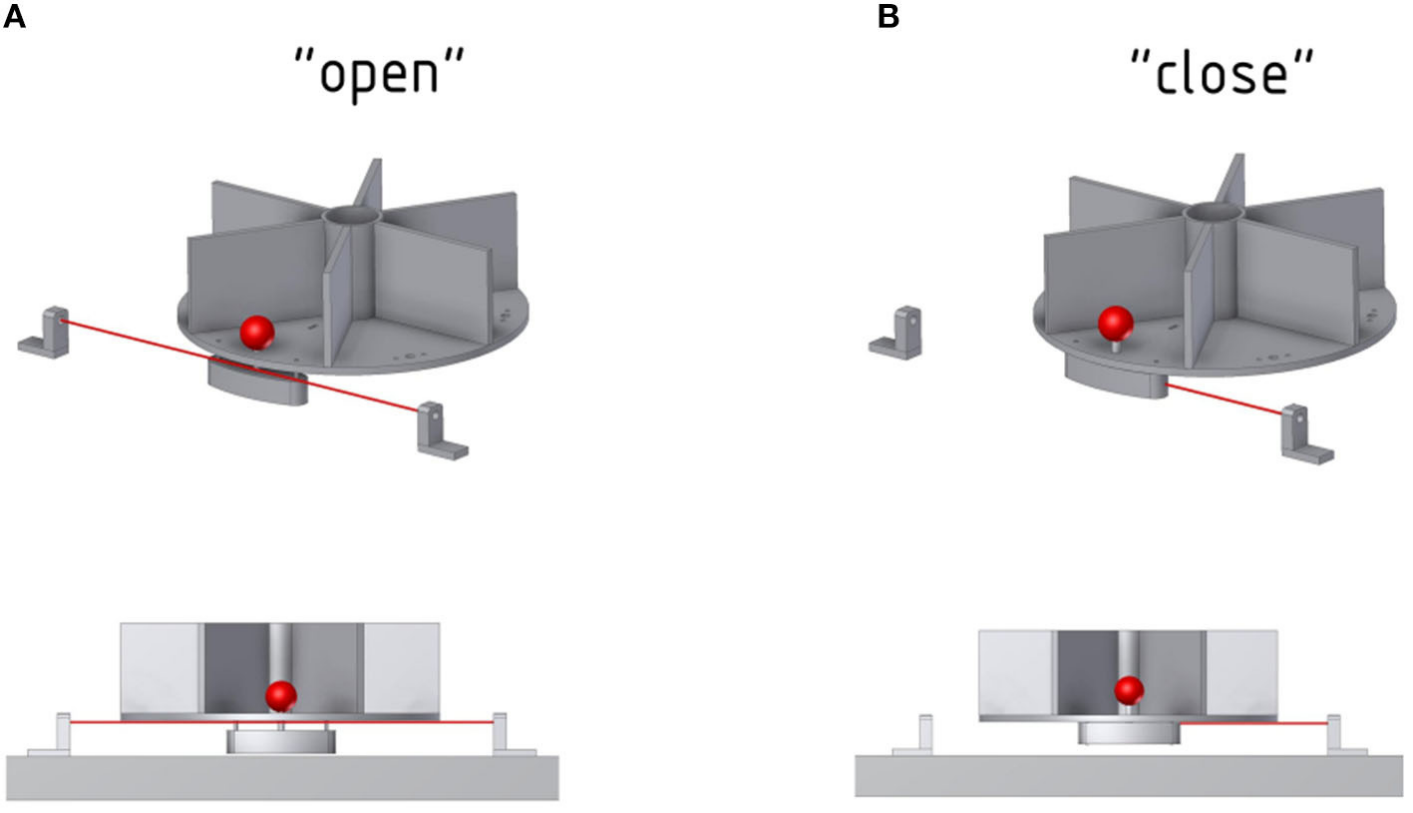
Function of the photoelectric Laser barrier. To determine whether an object has been lifted, a Laser and photoelectric element is placed below the object. **(A)** The Laser can pass through as long as the object is not lifted. **(B)** The counterweight of the front object will block the Laser once the object is lifted.

Each object was fitted with a counterweight below the object plate that was connected to the object with a carbon stick. The total weight of the object and counterweight was 120 g for all objects, independent of the object shape and size, to ensure a similar force required for lifting. Lifting height was 1 cm, forcing the monkey to actually lift the object, but with limited effort. Objects were placed near the outer border of the turntable disk (closest to the monkey) to avoid that the animal can rest his hand in front of the object. Furthermore, the balance point was placed as low as possible to ensure that the object is pulled down by gravity and cannot get stuck in the “lifted” position. The counterweight of each object doubled as trigger for a light barrier that was positioned below the object plate (see Figure 5). Whenever an object is lifted, the counterweight breaks the light-beam and the computer detects a successful object lift. To ensure a high sensitivity of the light barrier, a laser pointer with a small diameter was used to point on a light sensitive photo transistor.

The front of the setup consists of long barriers out of black plastic (see Figure 2B) that, together with small barriers next to each object, ensure that the monkey can see and interact only with the object currently facing him. This not only keeps the object presentation more stable, since only one object is within view at any time, but also doubles as a barrier to prevent the animal from interacting with the other objects during grasping or tactile object interaction. All plates are custom made out of black plastic sheets. A black plastic tube was fitted into the middle of the turntable to further obstruct view on other objects.

**Figure 6:**
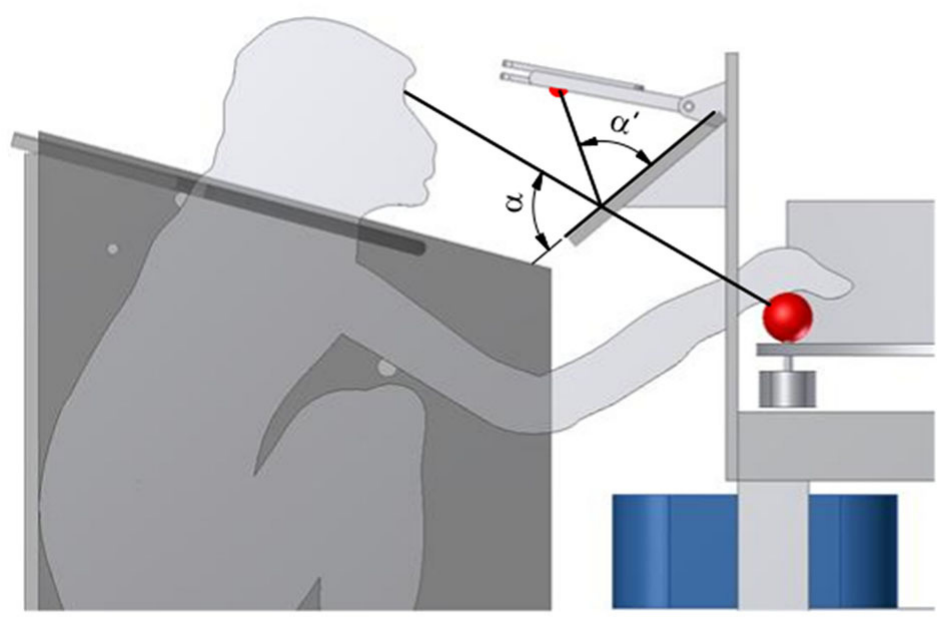
Projection of the cue LED light. To superimpose the red cue LED light onto the front object, from the monkey's perspective, a one-way mirror is placed, such that the angles α and α′ match. This avoids an attention split between the object and the cue LED.

A one-way mirror on a Plexiglas plate was mounted to the table using thick fiberglass bars, between the monkey and the object (see Figure 6). This mirror serves multiple functions. First, it reflects a red LED above the mirror onto the object. This cue LED is used by the monkey to determine when to interact with the object as either “explore cue” during tactile trials or “grasp cue” in both trial types. Projecting the LED light on the object prevents the animal from having to split attention between the position of the object and the cue LED, and also helps the animal not to move his eyes when the object is illuminated. Furthermore, this prevents the LED light being obscured by the monkey's arm. While the cue LED was usually turned off when the monkey's arm interacted with the object, this might be an important factor in other task paradigms. Second, the mirror could also be used for video-based eye tracking, without installing an additional eye-tracking mirror. Finally, the mirror serves as a barrier, making it harder for the animal to interfere with equipment, most notably the reward tube, located above the mirror. Additional LED lights could also be projected through the one-way mirror. We designed an LED plate featuring four LEDs that were placed in a reversed T shape that could be used to calibrate an eye tracker, and two additional yellow LEDs that were used to inform the animal of error trials.

3D printable STL files for the objects described above as well as more information on assembly are available at: https://github.com/NBL-DPZ/TurntableSetup.

### 2.4. Alignment of Collected Data

During the experiment, data from different sensors needed to be integrated. We utilized a capacitive-sensing touch button to detect whenever the animal was resting his hand at the start position, two light barriers (one to detect turntable rotation and one to detect lifting of the front object), and a data glove.

The setup was controlled by a NI PXI realtime System (National Instruments, Austin, TX, USA) with a clock rate of 1 ms and custom written LabView-Software. This included detection when the handrest button was pushed, the light barriers were triggered, and control of the rotation motor. Furthermore, the graphical user interface for the data glove (named KinemaTracks) was implemented in Matlab, as first described by Schaffelhofer and Scherberger (2012). As described above, two synchronized neural signal processors (NSPs; Cerebus systems, Blackrock Microsystems Inc., Salt Lake City, UT, USA) were used to record neural data and behavioral events. For the latter, the digital input port of the NSPs were used to record and synchronize all collected data from the three machines (NSPs, PXI-Box, and data glove PC).

The states of the sensors were encoded into numbers (e.g., 31 when the object was down, 32 when the object was lifted up) and written to the digital port of both NSPs as were numbers to identify the different epochs during this task. Due to usage of the same clock, this allowed to align neural data according to different epochs (or in theory even sensor states). A similar method was used to align additional data for the data glove. While the exact magnetic sensor positions remained on the dedicated data glove PC, a synchronization signal was sent to the NSP using the serial input every time a new data point was written onto the data glove PC, which was used to synchronize the clock of the data glove PC with that of the NSPs during offline analysis. Additional metadata were recorded during the intertrial state using the digital input port, such as time and date, which object was used during the current trial, and whether the trial was successful. This “tailer” always started and stopped with a specific sequence of numbers for easier data extraction during offline analysis. This way, all necessary information needed to synchronize the data of all sources was saved alongside the neural data on the recording PC.

### 2.5. Behavioral Paradigm

**Figure 7:**
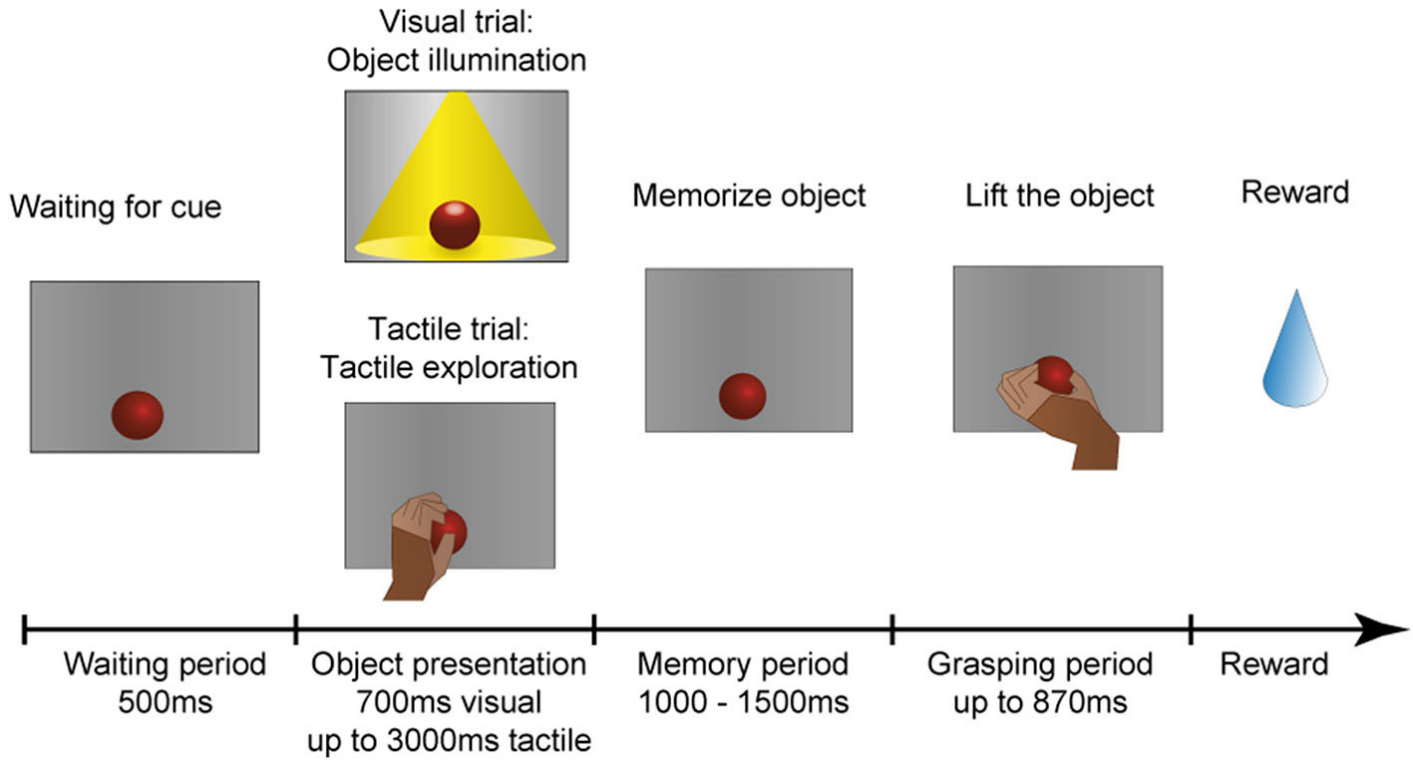
Task paradigm. After an object has arrived in front of the animal, it has to wait in the dark for an object presentation period, in which the animal could identify the object either visually or tactually. In the visual task, the object is illuminated for 700 ms. In the tactile task, however, the monkey remains in the dark and instead has to reach out, touch and briefly lift the object (maximal duration: 3,000 ms). The animal is then required to memorize the object for 1,000–1,500 ms before instructed to lift the object within 870 ms. All successful trials are rewarded with a fluid reward.

The monkey was trained in a delayed-grasping paradigm to grasp objects that he had either seen or touched beforehand. The main idea was to require the animal to either first look at an object and then grasp it in the dark, or to first touch and tactually explore the object in the dark before also grasping it in the dark. A diagram of this task is shown in Figure 7.

The animal is comfortably seated in a primate chair and sits in the dark during the whole experimental session. At the start of the experiment, a pseudo-random sequence of object presentation order is generated that ensures a uniform distribution of object occurrences, but prevents the animal from predicting the upcoming object in the next trial. The monkey can initiate a trial by placing his hand on the handrest button, which will start the turntable rotation. The turntable will stop at the appropriate object according to the aforementioned presentation sequence. Next, the object is presented either visually or tactually. For visual presentation, the object is illuminated for 700 ms, which instructs the monkey to sit still and simply look at the object. During tactile trials, however, the red cue LED above the object turns off as an explore cue (tactile trial: tactile exploration), which instructs the animal to reach out, touch, and lift up the object briefly within 3,000 ms to confirm haptic object exploration. This approach was chosen to encourage the monkey to actually interact with the object and to ensure that an appropriate grip for object lifting has been haptically explored. The animal then has to return to the handrest button during this object presentation period to ensure that the hand is always remaining still on the handrest button during the complete memory period, ensuring that the starting position of the hand is identical for visual and tactile trials. Afterwards, a memory period (memorize object) of random length (1,000–1,500 ms) occurs to avoid prediction of the grasp cue. As a last step, the red cue LED will turn off in both task conditions as a grasp cue, and the animal is required to quickly reach out, grasp and lift the object in the dark (within 870 ms). If this grasp and lift action is successful, the red cue LED turns on again and the animal has to return the hand to the handrest button to receive a reward (small amount of the animal's favorite juice). In case the animal made an error during any point of the task, two yellow error LEDs light up to indicate the error and the next trial starts after some short delay.

### 2.6. Data Analysis

#### 2.6.1. Movement Time Analysis

**Figure 8:**
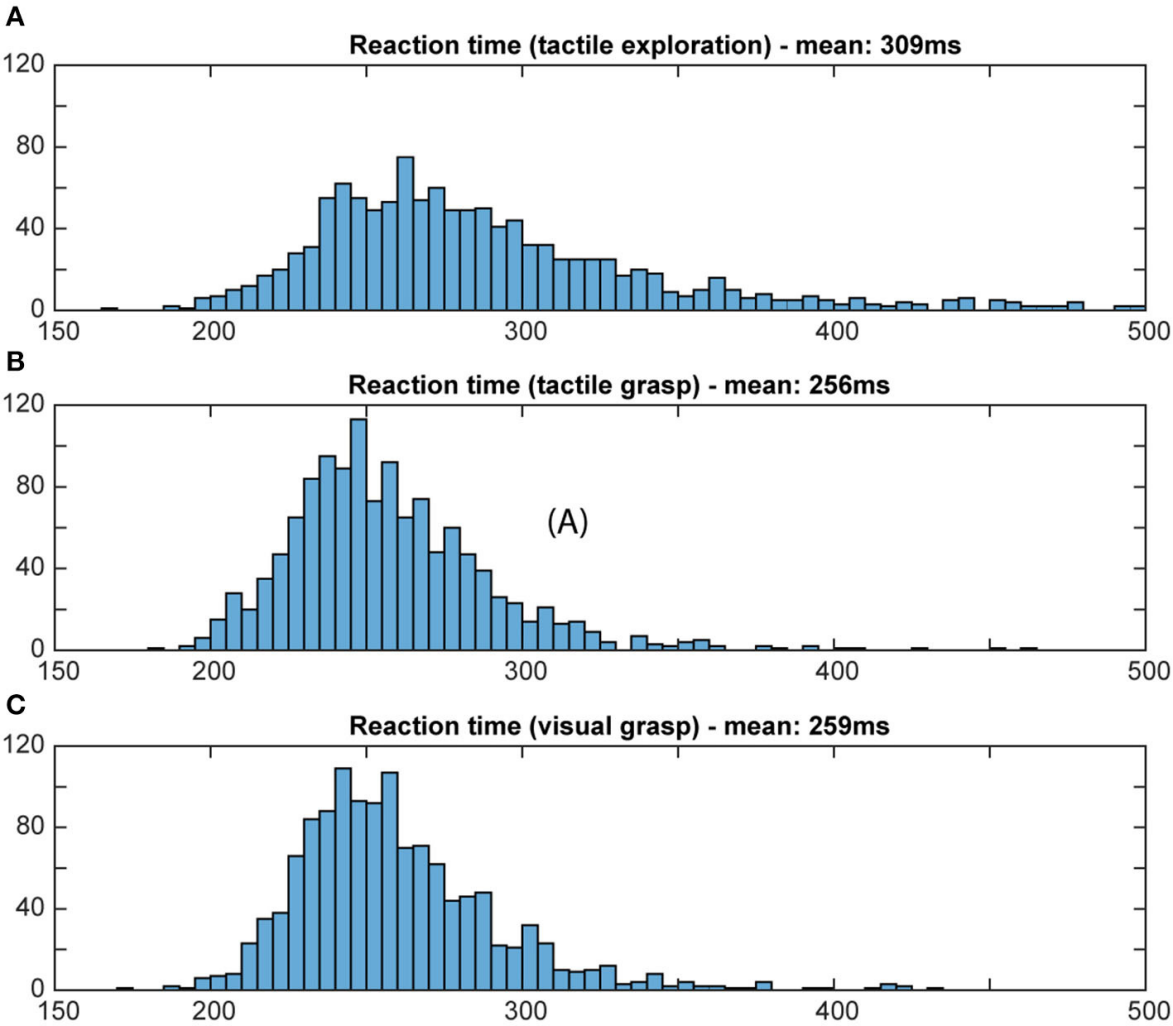
Distribution of reaction time. Histograms illustrate how often a certain reaction time occurred during the grasp period in the tactile object exploration **(A)** and during grasping in tactile **(B)** and visual trials **(C)**. Bin width: 5 ms; cut off at 500 ms. For tactile exploration **(A)**, the slightly higher mean and larger variation of reaction time suggests hesitation, e.g., due to the unknown object, and less preparedness. For both tactile and visual grasping **(B,C)**, a similar distribution of reaction time was observed, indicating that the animal obtained in both tasks sufficient object information during the object presentation period to plan an appropriate grasp.

**Figure 9:**
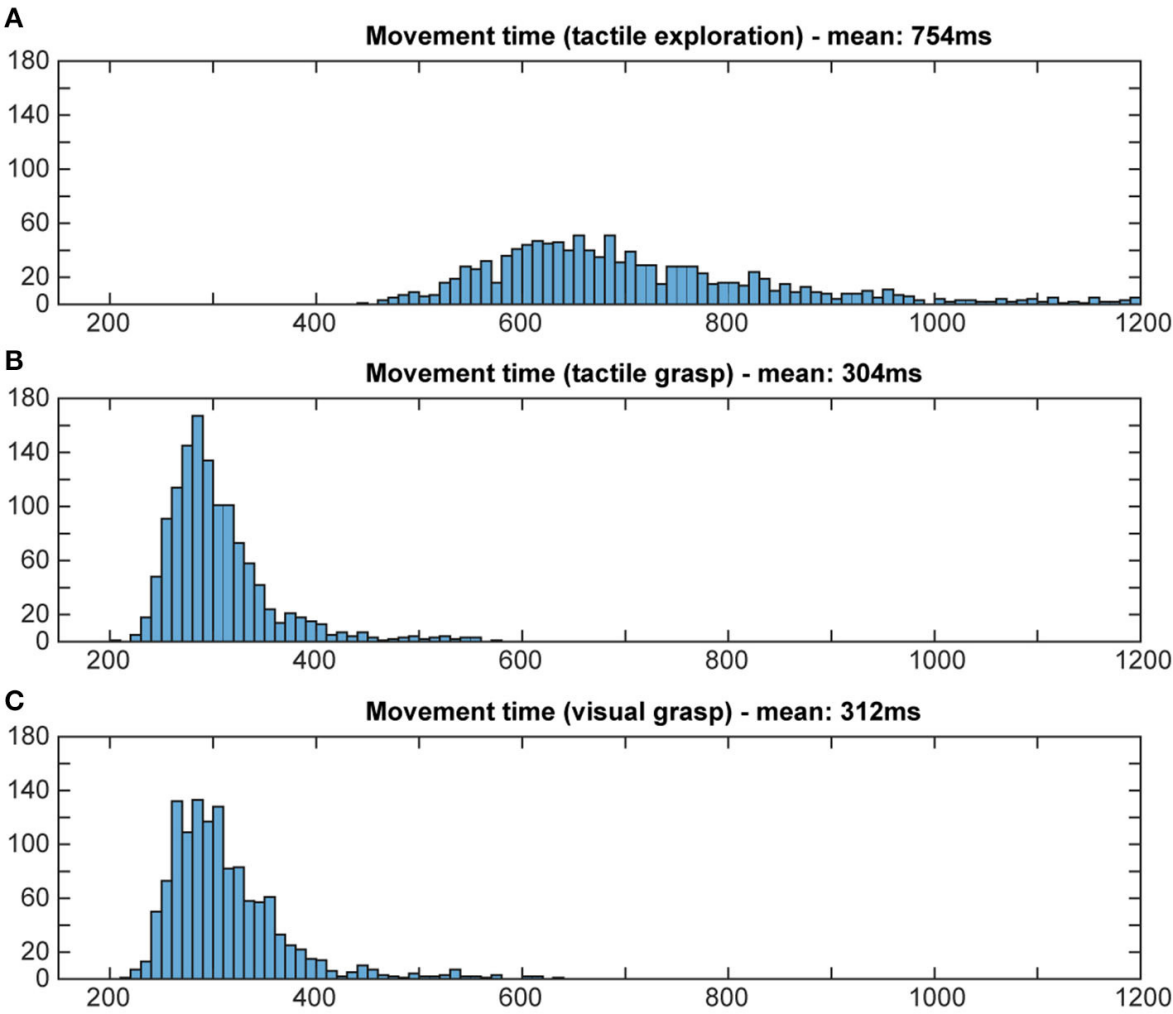
Distribution of movement times. Histograms illustrate how often a certain movement time occurred in both tasks. Bin width: 10 ms, cut off at 1,200 ms. During tactile exploration **(A)** the object was unknown, leading to multiple and varying grasp attempts and on average a much longer movement time. For grasp movement execution in the tactile **(B)** and visual grasps **(C)** a similar movement time can be observed, indicating that the animal was able to execute an appropriate grasp for the object based on the object information gathered from the object presentation period.

To evaluate whether or not the animal actually used object information during the final grasp period, we measured the reaction time and the movement time during visual and tactile trials, independently of object shape. A shorter movement and reaction time would indicate prior knowledge about the object, since an optimal grasp can be chosen right away (Michaels et al., 2018). If the monkey does not know the object's identity, he will need time to explore the object to find the best fit for his hand and therefore take longer. Reaction time was defined as the time between the appearance of the grasp cue and the release of the handrest button. Movement time was defined as the time when the monkey lost contact with the handrest button to explore (for tactile object exploration) or to grasp the object (for both grasp periods) until the object was fully lifted. Reaction and movement times were plotted as a histogram (bins width: 5 ms for reaction time; 10 ms for movement time; see Figures 8, 9).

#### 2.6.2. Neural Data Analysis

After raw data acquisition (see Implantation and neuronal signal acquisition, above), data was prepared for detection of spikes as previously described (Dann et al., 2016; Intveld et al., 2018; Michaels et al., 2018; Buchwald, 2020). The data was filtered with a median filter (window length: 3.33 ms) and the resulting signal subtracted from the raw signal. Then, a 4th order non-causal Butterworth filter (5,000 Hz) was applied as a low-pass filter (Butterworth, 1930). Channels where noise was already apparent during recording were excluded from the analysis. To remove common noise sources present in all channels (e.g., movement artifacts) a principal component analysis (PCA) artifact cancellation procedure was performed, as described in Musial et al. (2002). Only PCA dimensions with a coefficient larger than 0.36 (with respect to normalized data) were kept to avoid removing individual channels. Afterwards, data was spike-sorted using a modified version of Wave_Clus (Kraskov et al., 2004; Chaure et al., 2018). To demonstrate the feasibility of neural recording with this setup, two representative single units are presented below (see Results and Discussion).

**Figure 10:**
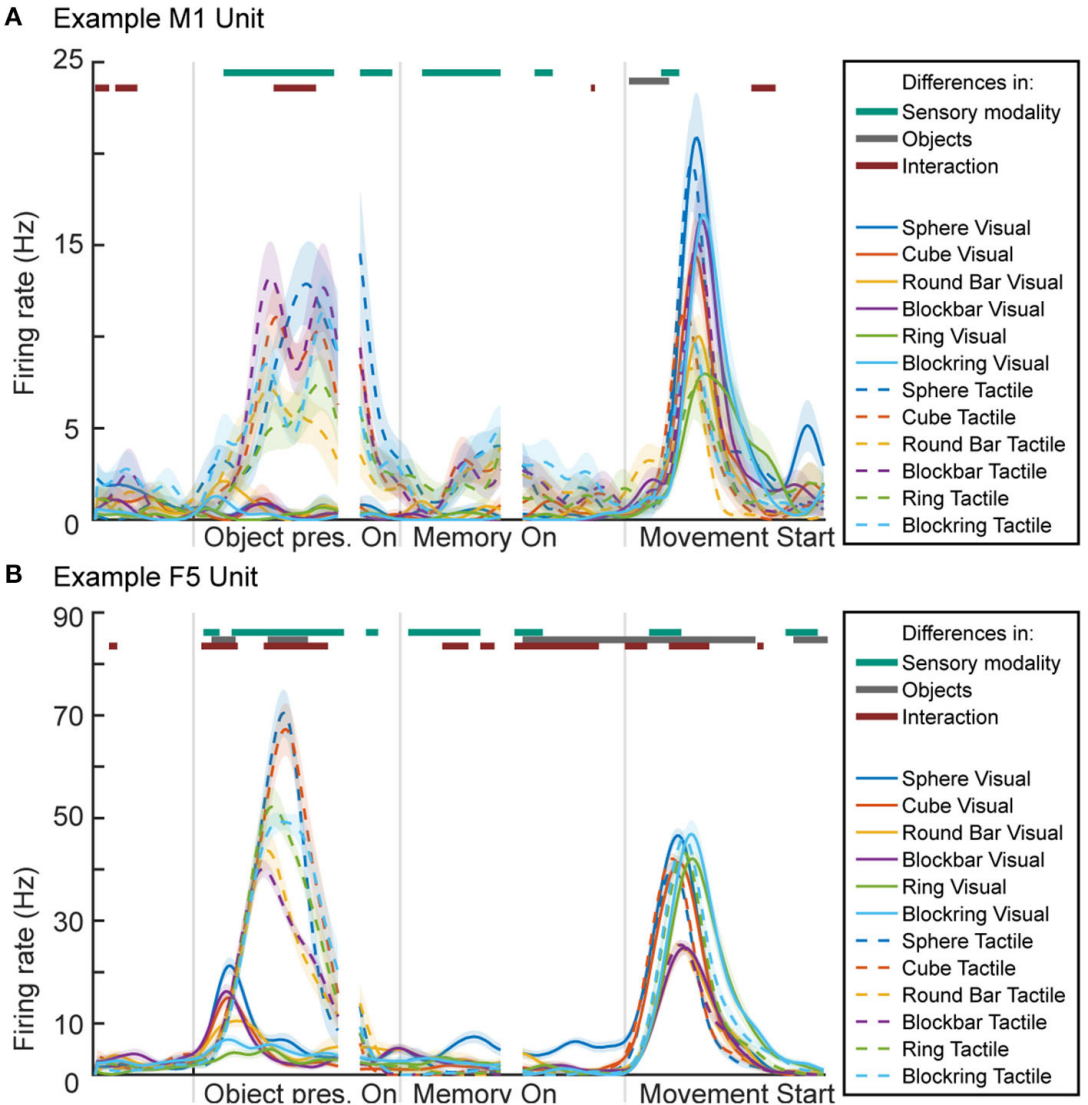
Peri-event time histogram of two example units from primary motor cortex (M1 unit) and premotor area F5 (F5 unit). **(A)** Activity during the two tasks of one M1 unit, showing higher activity during periods and task conditions that contain movement. **(B)** Activity of one F5 unit, showing activity during movement-related epochs as well as during object illumination. Colored dashed or solid lines: mean firing rate across trials for the visual task (solid lines) and tactile task (dashed lines) and the six objects: sphere, cube, round bar, block bar, ring, and block ring (different colors). Horizontal bars on top of the panels indicate periods with significant selectivity for sensory modality (vision vs. tactile; purple), object conditions (green), and interaction (blue); 2-way ANOVA with Bonferroni correction (*p* < 0.0001), see Methods.

For visualization of neuronal activity, we calculated a peri-event time histogram for each single unit (see Figure 10). For this, spike events were extracted and a Gaussian smoothing filter applied (SD: 50 ms) for every trial. The resulting firing rate curves where then aligned and averaged at three different time points, so that the influence of specific trial events can be better visualized. Data is presented 500 ms before object presentation (representing the baseline period where the animal is not engaged in any activity), followed by 700 ms after the onset of the illumination or explore cue LED, reflecting the neuronal response to seeing or touching the object. The second alignment point is the start of the memory period (time interval from 100 ms before until 500 ms after memory start), reflecting neural activity after the object presentation period ended (either stop of object illumination or a return to the handrest button) to ensure no more tactile object information is perceived and all movement ended. Third, data was aligned at movement start (500 ms before and 1,000 ms afterwards), reflecting grasp-related neural activity. To differentiate the 12 task conditions, six different colors were chosen for the objects while solid and dashed lines represent visual and tactile task trials, respectively.

To test for significant differences between task conditions, a 2-way sliding ANOVA (Analysis of variance) was conducted (sliding window: 100 ms, factors “objects” with six levels, “sensory modality” with two levels and “interaction” between factors) and Bonferroni-corrected for multiple comparison (*p*-value: 0.0001). Horizontal lines above the histogram indicate significant differences between the two sensory (visual vs. tactile) task modalities (purple), between objects (green), and significant differences caused by an interaction between objects and task modality (cyan).

## 3. Results and Discussion

### 3.1. Behavioral Analysis

Using this experimental setup, we have successfully trained one rhesus monkey in this grasping task paradigm, and furthermore have recorded behavioral and neural data from this animal, which we report in the following section.

For behavioral analysis of the recorded data, we analyzed the reaction and movement times during both tasks. We tested whether the monkey memorized the object information on both tasks or tried instead to guess the object. It can be assumed that the animal does recognize visually presented objects since seeing objects will give all information needed to grasp objects, especially for an animal that was trained on these objects beforehand and had the chance to familiarize itself with them (Gibson, 1958; Eimas, 1967; Dhawan et al., 2019).

Tactile object information however involves more effort, since the animal needs to move its hand around the object and match known tactile features of the object (Camponogara and Volcic, 2020). However, it was a priori unclear whether the monkey memorizes the object or instead prefers the easier but slower approach, to try out different grasps during the final movement epoch, until a fitting grasp is found for lifting up the object. In order to control for this, we determined the reaction time and movement time of the animal for all trials of both tasks during five recording sessions. Reaction time gives an insight into the preparedness of the animal, while movement time is influenced also by how quickly the object can be grasped and lifted, or whether grip adjustments were necessary (Michaels et al., 2018).

For reaction times, time between the occurrence of the grasp cue and the release of the handrest button, a broad distribution of movement times can be seen during the object presentation period of the tactile trials, when the animal is instructed to tactually explore the object, with a mean reaction time of 309 ms (standard deviation: 142 ms). During the grasp period of tactile and visual trials, however, we can see a more narrow distribution that looks very similar for visual and tactile trials, with a similar mean (mean: 256 and 269 ms, respectively, SD: 33 ms for both). This reflects very likely the state of knowledge of the monkey in these task epochs: during tactile exploration, the animal has no prior knowledge about the object, which might cause some hesitation and general unpreparedness. During tactile grasping, the monkey is then informed, since he has previously explored the object during the tactile exploration. During visual grasp, the monkey has already seen the object and knows which object he has to interact with, allowing him to prepare an appropriate grip. For movement time, a similar difference was observed for its distribution and mean (see Figure 9). During tactile exploration the movement times were generally longer and wider distributed with a mean of 754 ms (SD: 250 ms), reflecting that the animal did not know the object at this time point. Visual and tactile grasps showed a very similar distribution with a mean of 312 and 304 ms (SD: 58 and 52 ms), respectively, indicating that in both cases the animal was aware of the specific object it had to interact with. Reaction and movement time analysis therefore both confirm that the animal perceives the object identify prior to the final grasp of the object and independent of the sensory modality (vision or touch) and is able to use object information for the planning of a suitable grasp.

### 3.2. Neuronal Activity

To show the suitability of our setup also for neuronal recordings, we demonstrate the successful recording of neuronal activity from two neuronal units, one from primary motor cortex (M1) and one from premotor cortex (area F5) (see Figure 10). A lot is known about the activity of motor and premotor cortex during visually guided grasp conditions. The premotor cortex is mainly known as an area where movements are prepared, while motor cortex becomes active mainly during grasp execution (Fritsch and Hitzig, 1870; Penfield and Boldrey, 1937; Kakei et al., 1999; Hoshi and Tanji, 2000; Fluet et al., 2010; Schaffelhofer and Scherberger, 2016). In line with this past work, we found that activity in M1 and F5 were mainly active during epochs that contained movement, i.e., during tactile exploration of the object and during the actual grasp period in both tasks. This is illustrated in two example neurons from M1 and F5 (see Figure 10).

In both tasks (visual and tactile) the same six objects were presented. When comparing the activity in M1 and F5 for different objects during grasping, slight differences were observed in M1, while larger differences were visible in F5 (sliding ANOVA, Bonferroni corrected, *p* < 0.0001; see above: Data Analysis). In F5, objects that require a similar grasp (sphere and cube, round and blockbar, ring and blockring) elicit a similar firing rate during the object presentation epoch in the tactile task condition (dashed line), although significant differences between different objects can be found during this epoch (gray line above graph). When comparing visual (solid lines) vs. tactile conditions, we found no significant difference during movement, when the animal grasps the object, as signified by the lack of the top purple line above the graph during most of this period. In both areas the firing rate during grasping is similar for the same objects. During object presentation, however, significant differences are apparent (dark turquoise line on top of the graph). In both areas, the units remain at a lower firing rate during object illumination vs. tactile exploration. This was expected, since the tactile object presentation period contains movement, while the animal sits still during the object illumination in visual trials. In M1 this difference carries over during memory period, where a slightly higher firing rate can be observed, even though the monkey is no longer moving and has already returned to the handrest button. This is most likely remaining motor activity until the biological system had enough time to return to baseline (Evarts, 1968). This difference is significant during early but not late memory. In F5, the opposite is true, where object illumination seems to create a slightly higher firing rate in early memory. Both units show a short moment of significant difference after movement start, which might match findings in human trials, where haptically guided grasps (although by feeling an object in the other hand) lead to an earlier hand shaping but more cautious movement (Camponogara and Volcic, 2019).

### 3.3. Conclusion

In this paper, we presented a turntable setup that can be used to investigate hand movements in primates under different sensory conditions. The utilization of a motorized turntable with up to six objects allows randomized object presentation, where the monkey can work largely undisturbed from human interactions. Since turntables can be easily exchanged, even larger sets of objects can be presented in different blocks of trials, if needed by the experiment (e.g., see Schaffelhofer and Scherberger, 2016). Overall the number of objects can be scaled up as much as an experiment requires. While this setup was optimized for usage in non-human primates, it is also possible to use a modified version for human subjects, e.g., by scaling up the object size to allow for more natural hand movements.

We used this setup to demonstrate reaction and movement times between different task conditions, which can shed some light on how the animal uses object information to make informed decisions about the best hand grasp. Furthermore, with implants in multiple brain areas, we have demonstrated the suitability of this setup for investigating brain activity during different grasps (with different hand shaping) and different sensory conditions. This may serve for better understanding of how the brain integrates sensory information to generate meaningful movements.

## Data Availability Statement

The original contributions presented in the study are included in the article/supplementary material, further inquiries can be directed to the corresponding author/s. Additional files for reproduction of this setup are available at https://github.com/NBL-DPZ/TurntableSetup.

## Ethics Statement

The animal study was reviewed and approved by the Animal Welfare Division of the Office for Consumer Protection and Food Safety of the State of Lower Saxony, Germany (permit no. 14/1442 and 19/3132).

## Author Contributions

DB, SS, BD, MD, and HS conceived and designed the experiments. DB performed the experiments. DB and BD analyzed the results. DB, HS, and MD wrote the article. All authors provided comments and approved the manuscript.

## Conflict of Interest

The authors declare that the research was conducted in the absence of any commercial or financial relationships that could be construed as a potential conflict of interest.
